# Abnormal transcriptome-wide DNA demethylation induced by folate deficiency causes neural tube defects

**DOI:** 10.3389/fgene.2022.987210

**Published:** 2022-09-19

**Authors:** Shan Wang, Yubing Zeng, Pei Pei, Xuejia He, Fan Liu, Ting Zhang

**Affiliations:** ^1^ Beijing Municipal Key Laboratory of Child Development and Nutriomics, Capital Institute of Pediatrics, Beijing, China; ^2^ Capital Institute of Pediatrics-Peking University Teaching Hospital, Beijing, China; ^3^ Children’s Hospital Capital Institute of Pediatrics, Chinese Academy of Medical Sciences & Peking Union Medical College, Beijing, China

**Keywords:** DNA demethylation, DNA methylation, folate, neural tube defect, H3K27ac

## Abstract

Neural tube defect (NTDs) is one of the most common and serious fetal and neonatal birth defects. Neural tube closure (NTC) is an exquisitely coordinated process and this procedure is influenced by both genetic and environmental factor. Folic acid (FA) supplementation is an effective for prevention of a proportion of NTDs, however, the mechanism remains unclear. In this study, our data demonstrated genome-wide enrichment of 5-hydroxymethylcytosine (5hmC) modification on active transcriptional start sites (TSS) and decreased 5-methylcytosine (5mC) binding to TSS under folate deficiency in mESCs (mouse embryonic stem cells). Furthermore, folate deficiency promoted 5hmC enrichment enhancer histone 3 lysine 27 acetylation (H3K27ac) binding to Shh pathway genes in mESCs. Upregulation of Shh target genes was observed in mouse brain tissue under low levels of maternal serum folate, along with increased expression of 5-methylcytosine dioxygenase Tet1 levels. Taken together, we found that folate deficiency promoted DNA demethylation and enriched 5hmC through recruitment of H3K27ac to activate the Shh signaling pathway. These results suggest that the 5hmC modification increases concomitantly with a positive correlation to Shh gene expression in folate deficiency-induced mouse NTDs.

## Introduction

Neural tube defects (NTDs), including birth defects such as anencephaly, spina bifida, and cerebral palsy, are serious birth defects. An epidemiological study showed that 3% of newborns suffer from this disease worldwide. In recent years, the incidence of NTDs has decreased with nutritional improvement in China (Endalifer and Diress, 2020). There is incontrovertible evidence that NTDs are caused by environmental and genetic factors. FA, retinoic acid, inositol, and abnormal metabolism of other substances are environmental factors for this disease (Ebara, 2017; Greene et al., 2017; Cheng et al., 2019). Among these substances, FA is a major factor related to NTDs.

Deficiency of folate as a member of the vitamin B group leads to megaloblastic anemia. Folate mainly performs biological functions in the form of tetrahydrofolate. Its main role is to participate in the transfer of one-carbon units as a coenzyme in various biological reactions and affects DNA and histone methylation to alter gene expression (Field et al., 2018). A lack of methyl donors often reduces methylation levels in the genome. S-Adenosylmethionine (SAM) is a direct methyl donor and a severe lack of donor methyl groups results in hypomethylation throughout the genome. DNA methylation is mainly catalyzed by DNA methyltransferase (DNMT) 3a and DNMT3b during the embryonic stage, whereas DNA methylation is mainly catalyzed and maintained by DNMT1 during the cell division stage (Chen and Zhang, 2020). The proportion of SAM to S-adenosine homocysteine is a methylation index and a low ratio indicates intracellular hypomethylation. Reduced levels of DNA methylation can lead to the loss of certain chromosomes and impaired gene expression in an embryo. SAM as a methyl group donor *in vivo* has a direct effect on the transmethylated small molecule synthesis and the epigenetic modification mechanism of DNA and histone methylation, which has a vital role in maintaining the cell balance. Once this balance is disrupted, many developmental diseases occur in humans (Ebara, 2017; Field et al., 2018). For example, hippocampal atrophy, thinning of the microvascular system, apoptosis of neurons and glial cells, and an imbalance of neuronal cell proliferation and differentiation are closely related to abnormal embryonic development (Zhou et al., 2007; Yu et al., 2020; Dang et al., 2021).

DNA methylation as epigenetic information participates in many aspects of physiological and metabolic regulation during the mammalian life cycle. Under the effect of DNA, SAM is used as a methyl group. If a methyl group is covalently bonded to the fifth atom of the cytosine carbocyclic ring, it is called 5mC. This process converts 5mC into 5hmC, which is oxidized by translocated 10-11 dioxygenase (TET), resulting in DNA demethylation (Irwin et al., 2016). Ito et al. reported that 5hmC can be converted into 5-carboxylcytosine (5caC) and 5-formyloytosine (5fC) step-by-step *in vitro* (He et al., 2011; Ito et al., 2011). During embryonic brain development, genomic regions contain abundant 5hmC that undergoes demethylation and are widely regulated by DNA methylation and demethylation (Wen and Tang, 2014). DNA methylation is the predominant form in the adult brain. Its accumulation occurs during brain development in non-CG contexts (CH). Furthermore, CH methylation tends to accumulate rapidly in postnatally developing neurons and synchronizes with synaptogenesis (Lister et al., 2013).

Several epigenetic studies have been performed on human brain tissue, including regulation of DNA cytopyrimidine methylation and hydroxymethylation. Histone modifications are relevant to specific gene expression, gene silencing, and other chromatin states. Epigenetic regulation plays an important role in neuronal metabolism, maintenance of neuronal circuitry, and regulation of signal transmission. The distribution of 5hmC shows significant differences in various tissues of the human body. A previous study has suggested enrichment of 5hmC in brain tissue. Different tissues have the total levels of 5hmC with significant. The 5hmC content is highest in brain tissue, high in the liver, kidneys, and colon, and low in lungs, placenta, and heart (Li and Liu, 2011). In the heart, brain, and skeletal muscle, hypomethylation and/or hydroxymethylation of Notch receptor genes (*NOTCH1*, *NOTCH2*, *JAG2*, and *DLL1*) helps to control their expression, thereby contributing to cell regeneration and dynamic homeostasis (Terragni et al., 2014). Genome-wide and single-gene resolution atlas analysis showed that the 5hmC content in brain tissues increases with age, and age affects 5hmC levels in mitochondrial DNA in brain tissues. 5hmC is positively correlated to human cerebellum development. Studies in rhesus monkeys have verified that early maternal deprivation can get some regulations with 5hmC in the promoter regions of some genes associated with neurological functions and psychiatric disorders (Massart et al., 2014). 5hmC also has a strong regulatory function in neurons. Environmental factors closely influence the change. Several studies have demonstrated the role of the folate pathway in DNA methylation (Irwin et al., 2016; Liu et al., 2020; Araki et al., 2021). However, whether folate deficiency stress induces DNA hydroxymethylation and affects NTC-related genes causing NTDs remains unclear.

In this study, we found that folate deficiency increased expression of 5-methylcytosine dioxygenase and decreased DNA methylation transferase. Our data demonstrated genome-wide enrichment of 5hmC modification on active TSS and decreased 5mC binding to TSS under folate deficiency in mESCs. Furthermore, folate deficiency promoted 5hmC enrichment of enhancer H3K27ac binding to Shh pathway genes in mESCs. Upregulation of Shh target genes was observed in mouse brain tissue under low levels of maternal serum folate, along with aberrant Tet1 levels. Taken together, we found that folate deficiency promoted DNA demethylation and enriched 5hmC through recruitment of H3K27ac to activate the Shh signaling pathway. These results suggest that 5hmC modification increases concomitantly with a positive correlation to Shh gene expression in folate deficiency-induced mouse NTDs.

## Results

### Different expression of DNA demethylation enzymes in brain tissue neural tube defect

**FIGURE 1:**
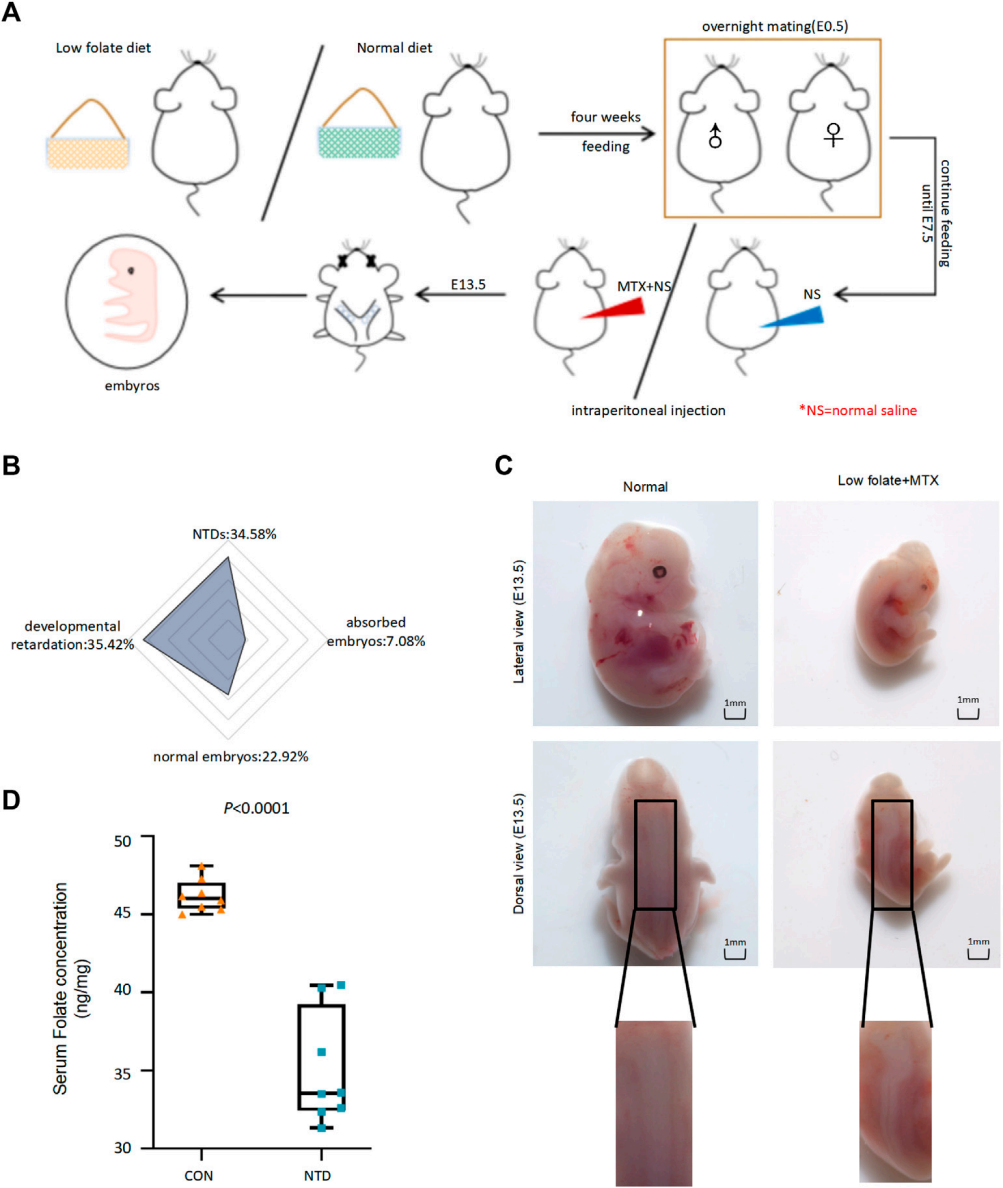
**(A)**The flow chart demonstrated modeling of NTD mice construction process. **(B)** The phenotype of embryos in low folate + MTX group was shown by radar map divided into NTDs (34.58%), absorbed embryos (7.08%), developmental retardation (35.42%), normal embryos (22.92%). **(C)** The phenotype of normal and low folate + MTX induced embryos from E13.5. **(D)**The maternal serums were detected in the pregnant mice with NTDs and control group (CON).

**FIGURE 2:**
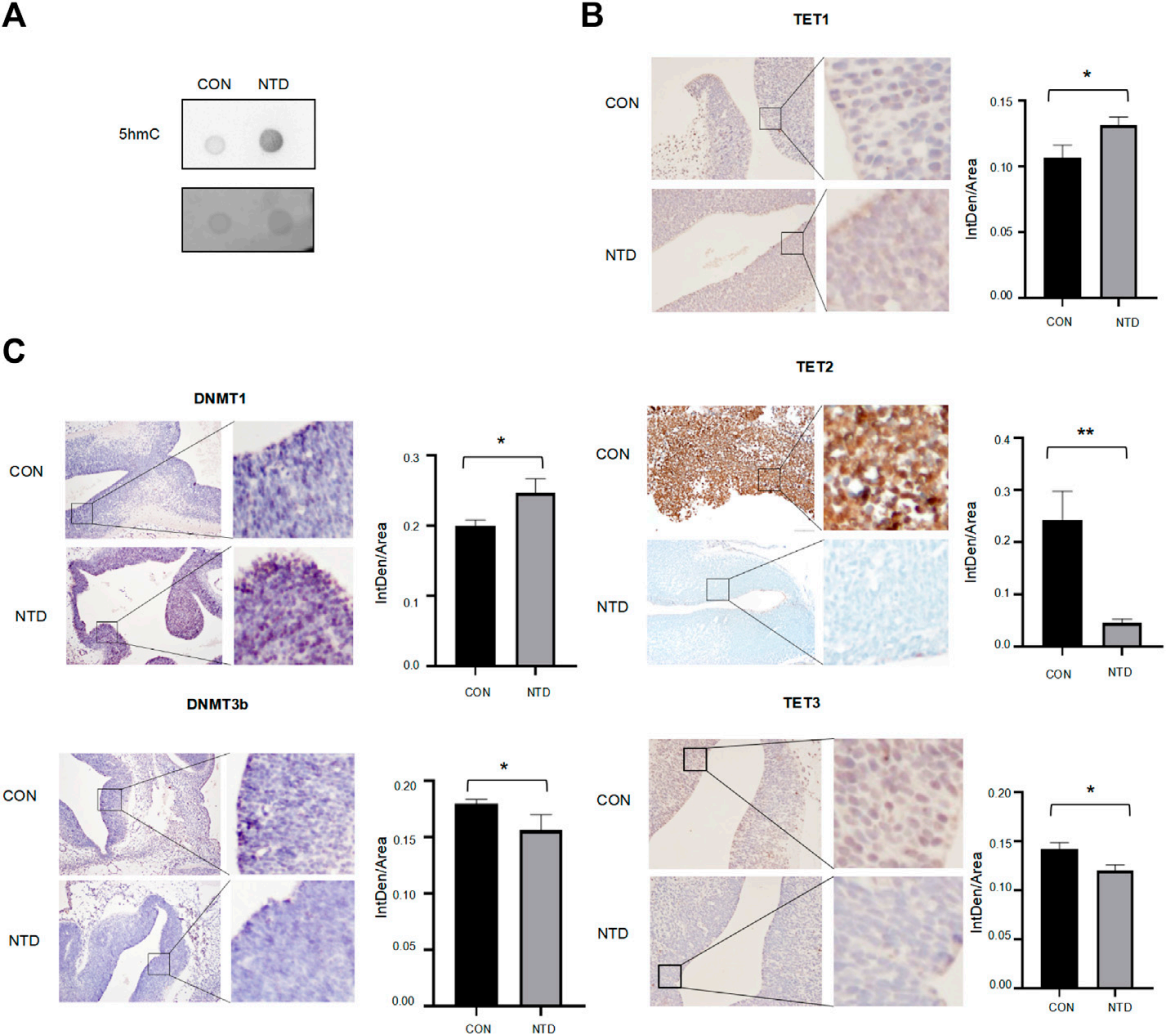
**(A)** Image of 5hmC-specific dot blot. **(B,C)** Representative images from immunohistochemical staining of TET1, TET2, TET3, DNMT1, DNMT3B in the cranial neural tissue from E13.5. TET1, TET2, TET3, DNMT1, DNMT3B expression scores is shown as histogram, with the horizontal lines representing the median. Data are mean ± SD. (*n* = 3), *p < 0.05, **p < 0.01, ***p < 0.001, by Student’s t test.

FA affects nucleotide synthesis, which may be involved in NTDs. Here, we established an NTD mouse by intraperitoneal injection of Methotrexate (MTX) (1.5 mg/kg) at embryonic day 7.5 and a low folate diet. The modeling of NTD mice construction process was shown in Figure 1A. As the amount of MTX decreased, the specific rate of normal fetal mice increased, and the specific rate of fetal aspiration decreased. When the dose of MTX was 1.50 mg/kg, the survival rate of the embryo was 22.92%, the rate of absorption dropped to 7.08%, and the rate of malformation was the highest deformity rate of 34.58% in low folate diet plus MTX group and the phenotype of NTDs was mainly spina bifida (Figure 1B). The failure of closure at the level of the hindbrain/cervical boundary at this stage leads to craniorachischisis (Figure 1C). We assessed the concentration of folate in maternal serum in normal group (CON) and low folate seed with MTX-induced NTD group (NTD). Folate levels were significantly decreased in the maternal serum (Figure 1D), suggesting that low folate diet and MTX resulted in folate deficiency affecting NTDs in fetuses during early pregnancy. Next, we quantified the total amount of 5hmC in both normal and NTDs brain tissues with a standard dot-blot assay. We found significant inductions in NTDs compared to the control (Figure 2A). Furthermore, we examined different DNA demethylation levels, including TET1, TET2 and TET3 and DNA methylation levels including DNMT1, DNMT3b in three cranial neural tissue samples and matched normal tissues by IHC analysis. The staining of total TET1 was increased and TET2, TET3 was decreased in mouse NTD samples compared with normal tissues (Figure 2B). The staining of total DNMT1 was increased and DNMT3b was decreased (Figure 2C). These results indicate that low folate diet and MTX alter DNA demethylation transferases involved in DNA demethylation.

### Increased expression of 5-methylcytosine dioxygenase in folate-deficient mouse ESCs

**FIGURE 3:**
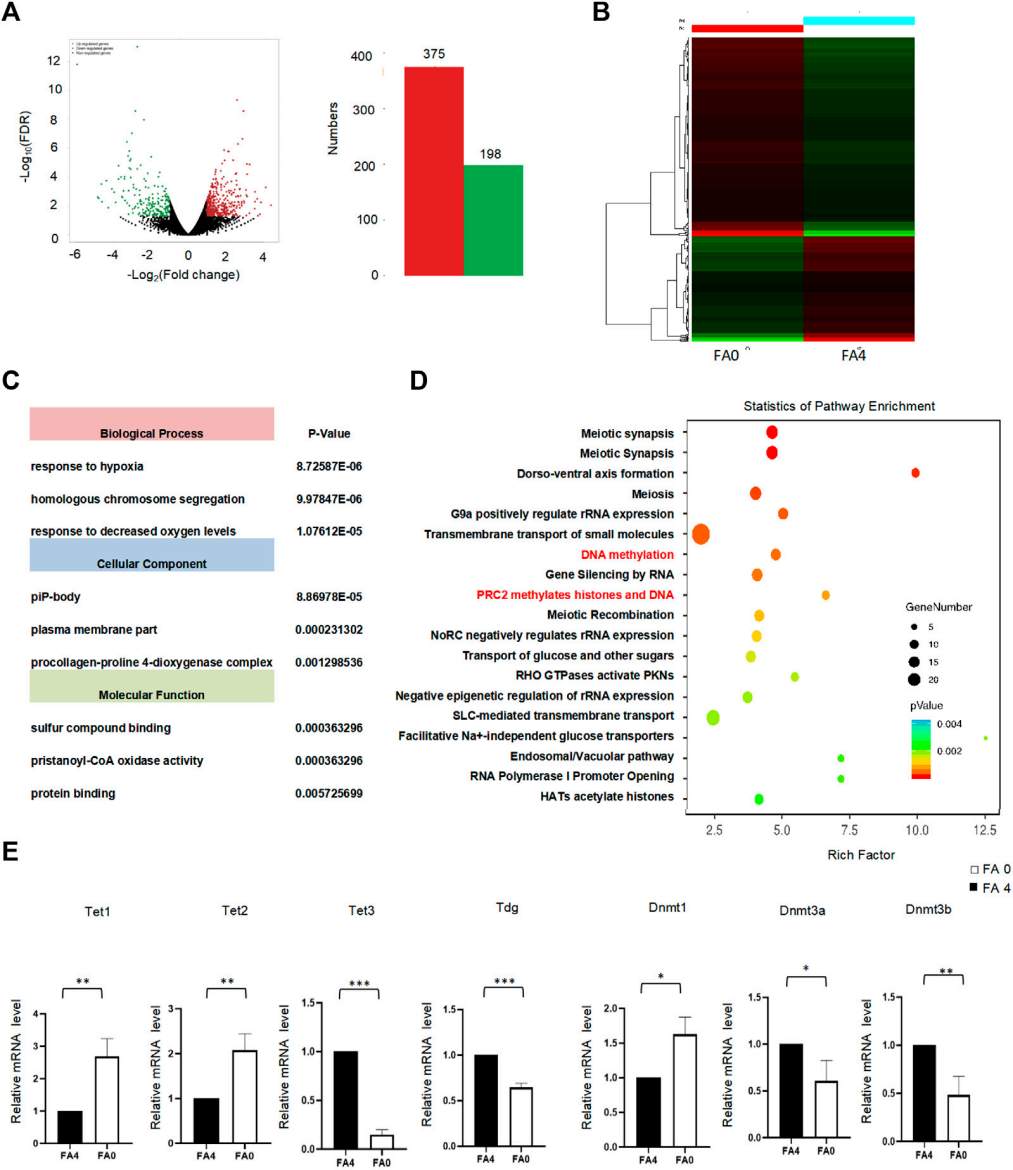
**(A)** FA (0 mg/L) vs. FA (4 mg/L) in mESC. The total RNA was analyzed using RNA-seq. A total of differentially expressed genes showing differential expression were identified in the APN-treatment group (*p* < 0.05). **(B)** Unsupervised hierarchical clustering of FA (0 mg/L) vs. FA (4 mg/L) in mESC. **(C)** Bar chart of the differentially expressed genes organized by enriched BP, CC, and MF, as determined with the Database for Annotation, Visualization, and Integrated Discovery (DAVID). **(D)** KEGG enrichment analysis (Fisher’s exact test; *p* < 0.05). The most popular paths are shown in each category. **(E)** The mRNA level of DNA methylases and demethylases was assessed by RT-qPCR between FA (0 mg/L) vs. FA (4 mg/L) in mESC. GAPDH was used as control. *p < 0.05, **p < 0.01, ***p < 0.001.

To evaluate the effect of folate deficiency in mouse ESCs, we performed RNA-seq of mouse ESCs with normal folate (4 mg/L) (FA4) and folate deficiency (0 mg/L) (FA0). We identified differentially expressed genes in folate-deficient cells. A total of 375 genes showed increased expression and 198 showed decreased expression (Figure 3A; Supplementary Table S1). A heatmap showed that folate deficiency induced broad modulation of genes in mouse ESCs (Figure 3B). To investigate the effect of folate deficiency on host functions, GO analysis revealed that these differentially expressed genes were enriched in GO terms multiple biological processes, molecular functions, and cell component as shown in Figure 3C (Supplementary Table S2). The *p*-value of Fisher’s exact test showed that the top three biological process groups of differentially expressed genes were response to hypoxia, homologous chromosome segregation, and response to decreased oxygen levels. The top three cellular components of these genes were piP-body, plasma membrane part, and procollagen-proline 4-dioxygenase complex. The top three molecular functions relevant to these proteins were sulfur compound binding, pristanoyl-CoA oxidase activity, and protein binding. Further KEGG pathways of developmental biology included signaling DNA methylation and PRC2 methylates histones and DNA (Figure 3D, Supplementary Table S3). Folic acid as a carbon unit provides methyl groups directly or indirectly, thereby affecting methylation of DNA and histones and altering gene expression. DNA methylation during the embryonic stage is mainly catalyzed by DNA methylation transferases such as DNMT3a and DNMT3b, while DNA methylation in the cell division stage is mainly catalyzed and maintained by DNMT1. 5mC is oxidized to 5hmC by TET family of proteins, which is known as DNA hydroxymethylation. 5hmC does not only bring its superiority into the demethylation process, but also participates in the regulation of gene expression. TET family proteins are iron-divalent dioxygenase containing an α group ketoglutaric acid, including TET1, TET2, and TET3. To determine whether folate deficiency specifically regulated DNA methylation transferase and DNA hydroxylase genes, the expression of a series of reported DNA methylation transferase and DNA hydroxylase-related genes was measured by quantitative PCR, including DNA methylation transferase *Dnmt3a*, *Dnmt3b*, and *Dnmt1*, and DNA hydroxylase-related genes *Tet1*, *Tet2*, *Tet3*, and *Tdg*. Folate deficiency increased the mRNA levels of DNA hydroxylase-related genes *Tet1*, *Tet2* and the mRNA levels of DNA methylation transferases *Dnmt1*, but reduced the mRNA levels of DNA methylation transferases *Dnmt3a* and *Dnmt3b*, and DNA hydroxylase-related genes *Tet3*, Tdg in mESCs (Figure 3E). Thus, folate deficiency increased expression of 5-methylcytosine dioxygenase and decreased DNA methylation transferases involved in DNA demethylation.

### Folate deficiency contributes to transcriptional start sites activation through DNA demethylation in mouse embryonic stem cells

**FIGURE 4:**
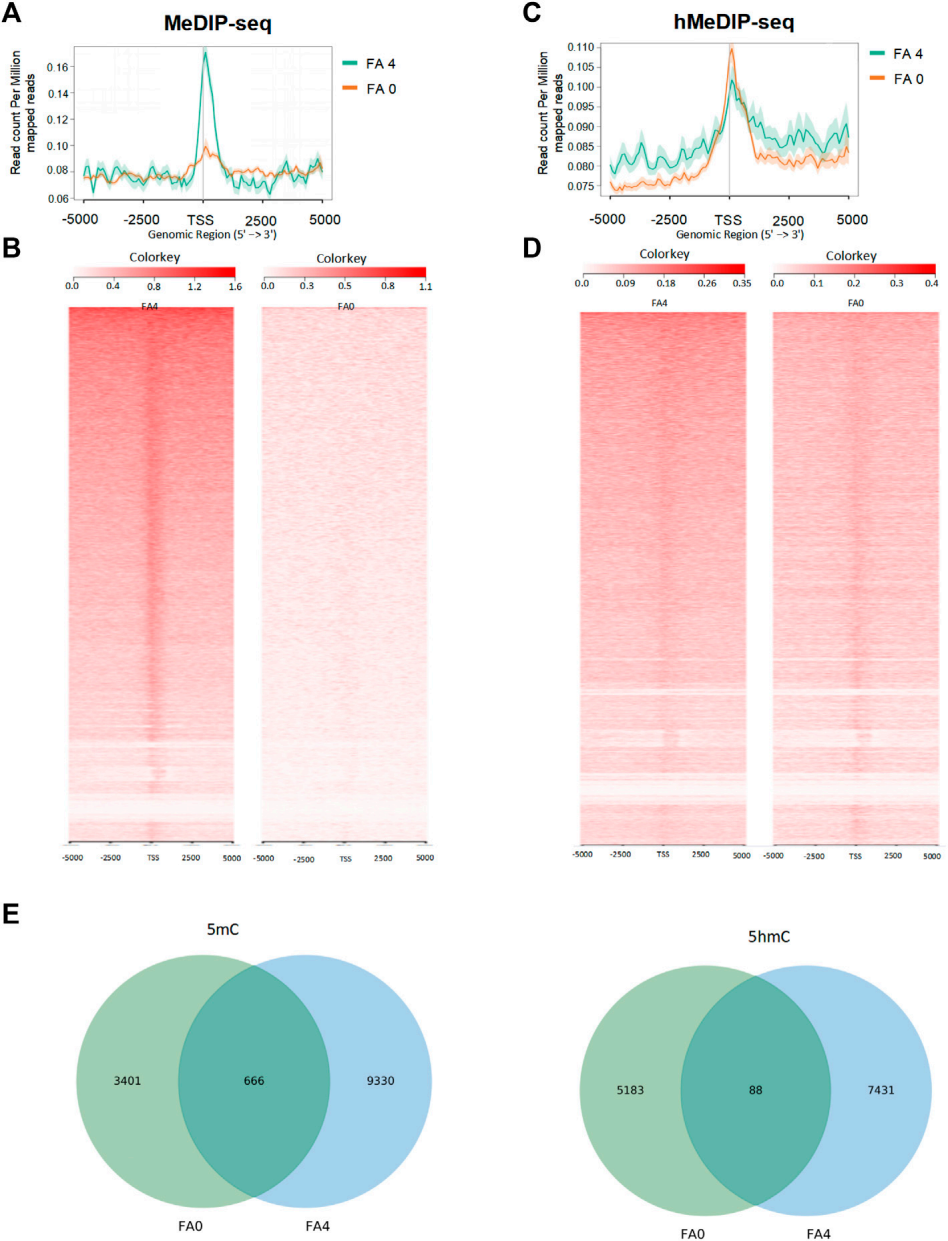
Genomic landscapes of 5mC and 5hmC were detected by MeDIP and hMeDIP deep sequencing in FA (0 mg/L) vs. FA (4 mg/L) mESC. **(A,C)** Average intensity plots of 5mC and 5hmC enrichment aggregated around TSSs. **(B,D)** Heatmap format ranked by read intensity. **(E)** The Venn diagrams showed the enrich-regions of 5mC and 5hmC by MeDIP and hMeDIP.

To further explore the importance of 5mC and 5hmC during neural system development, we compared the genome-wide distribution of 5mC and 5hmC. We performed MeDIP-seq for 5mC and hMeDIP-seq for 5hmC to map their genomic landscapes between normal folate and folate-deficient mouse ESCs. MeDIP-seq of 5mC target genes showed decreased enrichment levels of 5mC in folate-deficient mESCs compared with those in controls (Figure 4A, Supplementary Table S4). hMeDIP-seq of 5hmC target genes revealed increased enrichment levels of 5hmC in folate-deficient mESCs compared with those in controls (Figure 4C, Supplementary Table S5). Both MeDIP-seq and hMeDIP-seq analyses showed a remarkable overall change in 5mC and 5hmC near transcription start sites under folate deficiency. Heatmap clustering of the 5hmC peak distributions 5-kb upstream and downstream of TSSs revealed that higher 5hmC signals and lower 5mC signals in the folate-deficient group compared with those in the control group (Figures 4B,D). This suggested that folate deficiency induced a dramatic change in 5mC and 5hmC peak signals. In both 5mC and 5hmC groups, more than 50% of the peaks resided in intergenic regions and fewer than 30% of the peaks appeared in the gene bodies of annotated genes (Supplementary Figure S1). Further, we compared enriched regions separately for 5mC and 5hmC in control group and folate-deficient group. Results suggested that enriched regions of 5mC and 5hmC have significant changes between folate-deficient mouse ESCs and control mESCs (Figure 4E). Taken together, our data demonstrated genome-wide enrichment of the 5hmC modification on active TSSs and decreased 5mC on repressed TSSs under folate deficiency in mESCs.

### Enrichment clustering analysis of methylated and hydroxymethylated genes in folate-deficient mouse embryonic stem cellss

**FIGURE 5:**
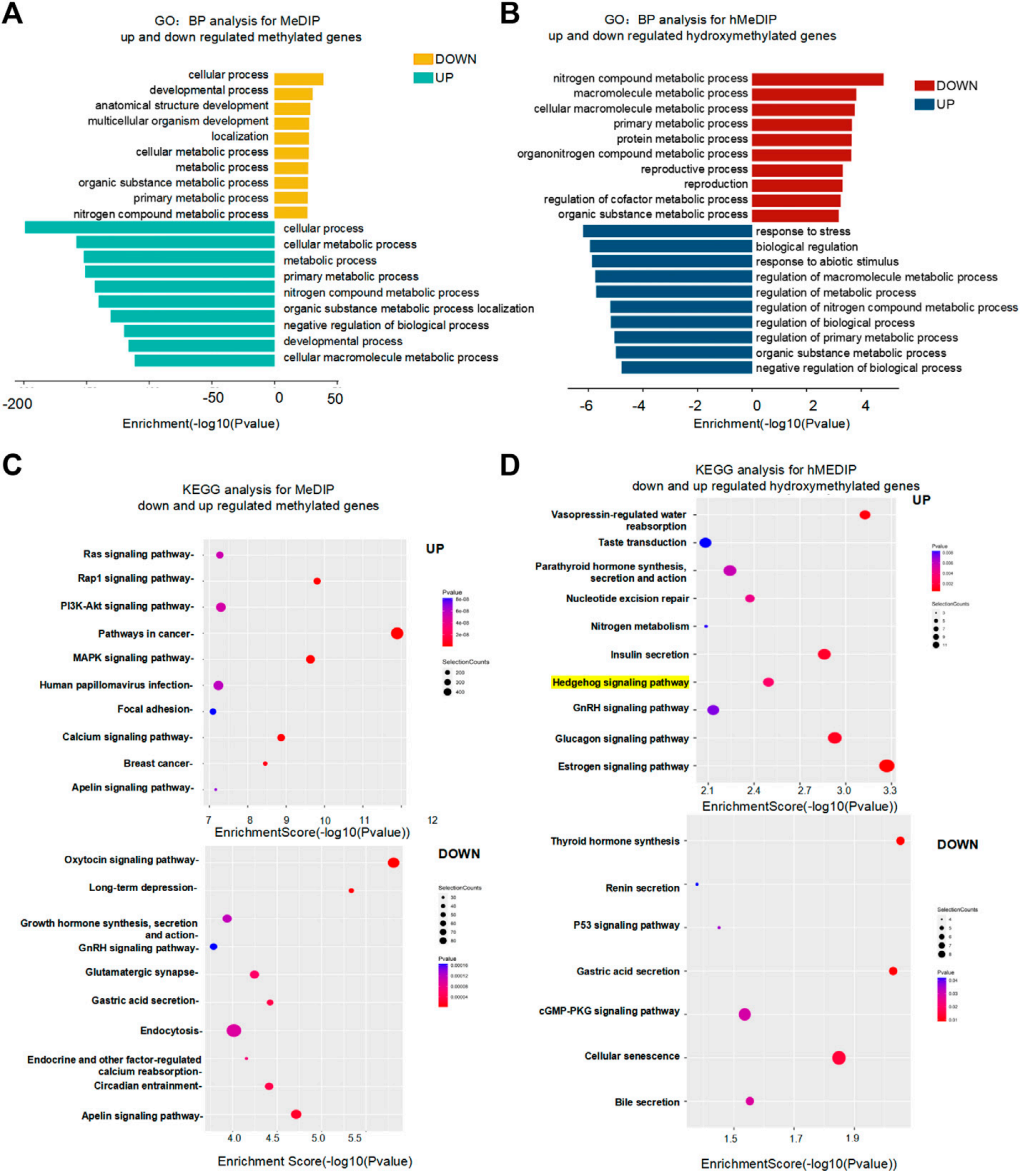
**(A,B)**. Functional network of enriched genes with MeDIP-Seq and hMeDIP-Seq peaks in FA (0 mg/L) vs. FA (4 mg/L) mESC. DAVID method was used to do functional annotation clustering for biological process annotations of upregulated and downregulated methylated or hydroxymethylated genes with MeDIP-Seq and hMeDIP-Seq. **(C,D)** KEGG enrichment analysis of upregulated and downregulated methylated or hydroxymethylated genes in FA (0 mg/L) vs. FA (4 mg/L) mESC. (Fisher’s exact test; *p* < 0.05). The most popular paths are shown in each category.

We next analyzed genes containing altered 5mC and 5hmC peaks for more insights into 5mC and 5hmC under folate deficiency in mouse ESCs. The results showed that 61,194 genes were differentially methylated genes between normal folate and folate-deficient mouse ESCs, of which 33,992 were methylated and 27,202 were unmethylated (Supplementary Table S6). Gene Ontology Biological Process analysis showed that the unmethylated genes participated in cellular and developmental processes, and methylated genes were mostly involved in the cellular metabolic process (Figure 5A, Supplementary Tables S7 and S10). Results of GO Molecular Function and GO Cell Component analyses are shown in Supplementary Tables S8, S9, S11, S12. A total of 11,552 genes were differentially hydroxymethylated between normal folate and folate-deficient mouse ESCs, of which 5,508 genes were methylated and 6,044 genes were unmethylated (Supplementary Table S13). GO Biological Process analysis revealed that low hydroxymethylated genes were involved in nitrogen compound and macromolecule metabolic processes, and hydroxymethylated genes were mostly involved in the response to stress and biological regulation (Figure 5B, Supplementary Tables S14, S17). Molecular Function and Cell Component analyses of hydroxymethylated genes are shown in Supplementary Tables S15, S16, S18, S19. KEGG analysis revealed that the top three terms with enriched unmethylated genes were the Oxytocin signaling pathway, Long-term depression, and Apelin signaling pathway, while those of methylated genes were the Pathways in cancer, Rap1 signaling pathway, and MAPK signaling pathway. The top three terms with enriched non-hydroxymethylated genes were Thyroid hormone synthesis, Gastric acid secretion, and Cellular senescence, while those of hydroxymethylated genes were the Estrogen signaling pathway, Vasopressin-regulated water reabsorption, and Glucagon signaling pathway (Figures 5C,D, Supplementary Tables S20–S23). Interestingly, KEGG analysis also showed that hydroxymethylated genes were involved in the NTD-related Hedgehog signaling pathway and non-hydroxymethylated genes were involved in the p53 signaling pathway. These results suggested that hydroxymethylated genes regulated NTD-related pathways.

### Folate deficiency promotes 5hmC binding to Shh pathway genes in mESCs

**FIGURE 6:**
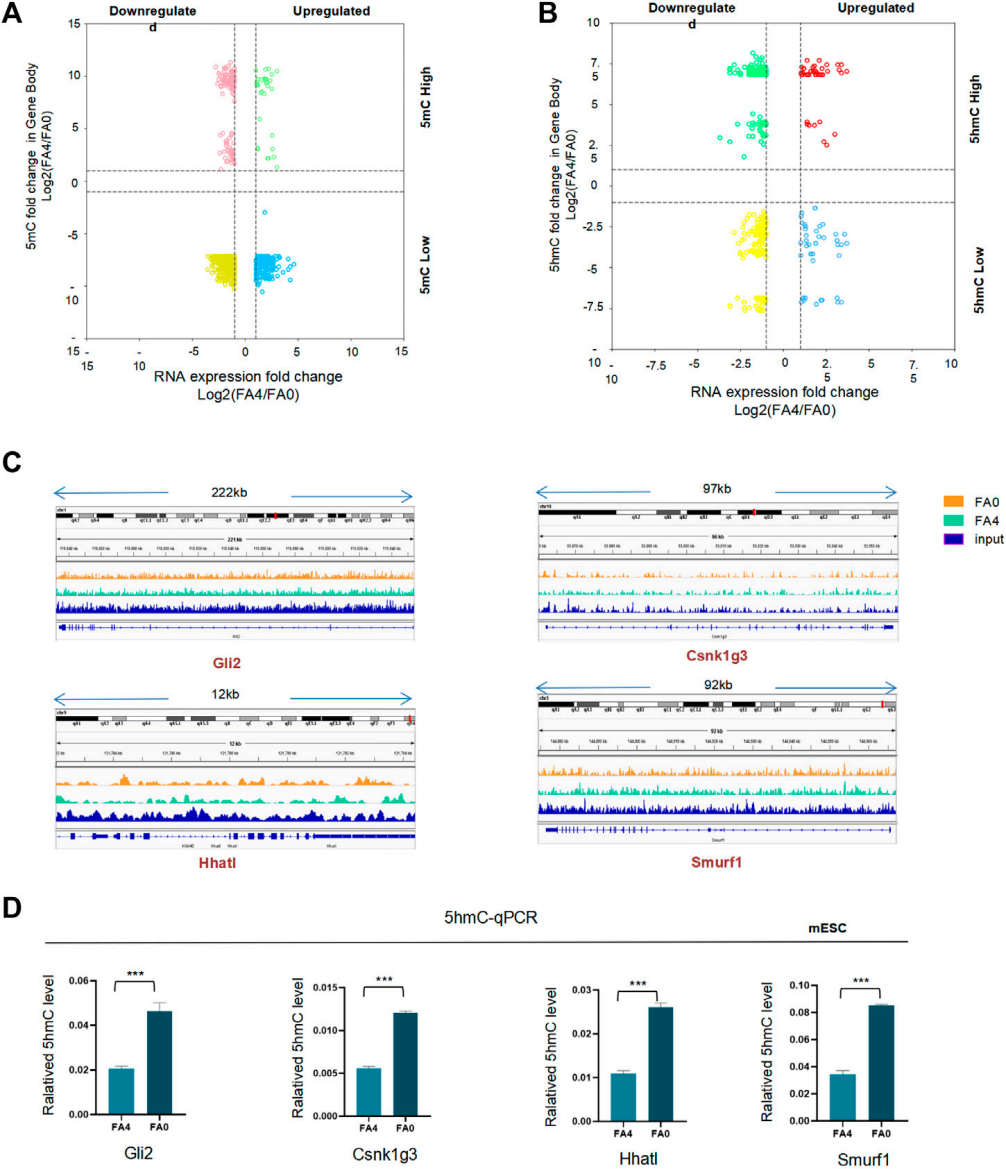
(A.B) Four quadrant diagram of differentially methylated **(A)** and demethylated **(B)** peaks at gene bodies and differentially expressed genes (FA4 vs. FA0, *p* < 0.05). The *x*-axis is Log2 fold change of RNA expression level, and the *y* axis is the difference in DNA demethylation. Dashed lines indicate a twofold difference. **(C)** hMeDIP-Seq density profiles for FA4 and FA0 at the genes (Gli2, Hhatl, Smurf1, Csnk1g3) in mESC by IGV. **(D)** The 5hmC level of the Shh-related to genes (Gli2, Hhatl, Smurf1, Csnk1g3) expressed significant differences in FA (0 mg/L) vs. FA (4 mg/L) mESC. *p < 0.05, **p < 0.01, ***p < 0.001.

We next examined the connection by assimilating RNA-seq data into methylated/hydroxymethylation and gene expression in promotor regions (Figures 6A,B, Supplementary Tables S24, S25). We also analyzed the percentages were comparable in promoter regions that were differentially hydroxymethylated with 38.07% of genes downregulated and 61.93% of genes upregulated (Supplementary Table S25). These results demonstrated that DNA hydroxymethylation was proportional to gene expression in the presence of folate deficiency. Shh genes had higher hydroxymethylation in gene body regions in the absence of folic acid compared with those in controls. For example, *Gli2*, *Hhatl*, *Smurf1*, and *Gsnk1g3* are Shh-related genes. 5hmC profiles of representative Shh-related genes showed high 5hmC levels under folate deficiency (Figure 6C and Supplementary Table S25). 5hmC profiles of some other core genes (*Boc*, *Gas1*, *Cdon*, *Smo*, *Shh*, *Gli1*, *Gli3*) in Shh pathway were shown in Supplementary Figure S2. To ensure the reliability of this genome-wide phenomenon, we performed hMeDIP-qPCR of Shh genes. As shown in Figure 6D, hMeDIP-PCR revealed that binding of these Shh-related genes (*Gli2*, *Hhatl*, *Smurf1*, and *Gsnk1g3*) to 5hmC was significantly increased upon folate deficiency. Thus, folate deficiency promoted upregulation of 5hmC and subsequently increased binding of 5hmC to the promoters of *Gli2*, *Hhatl*, *Smurf1*, and *Gsnk1g3*.

### Folate deficiency promotes H3K27ac binding to Shh genes in mESCs

**FIGURE 7:**
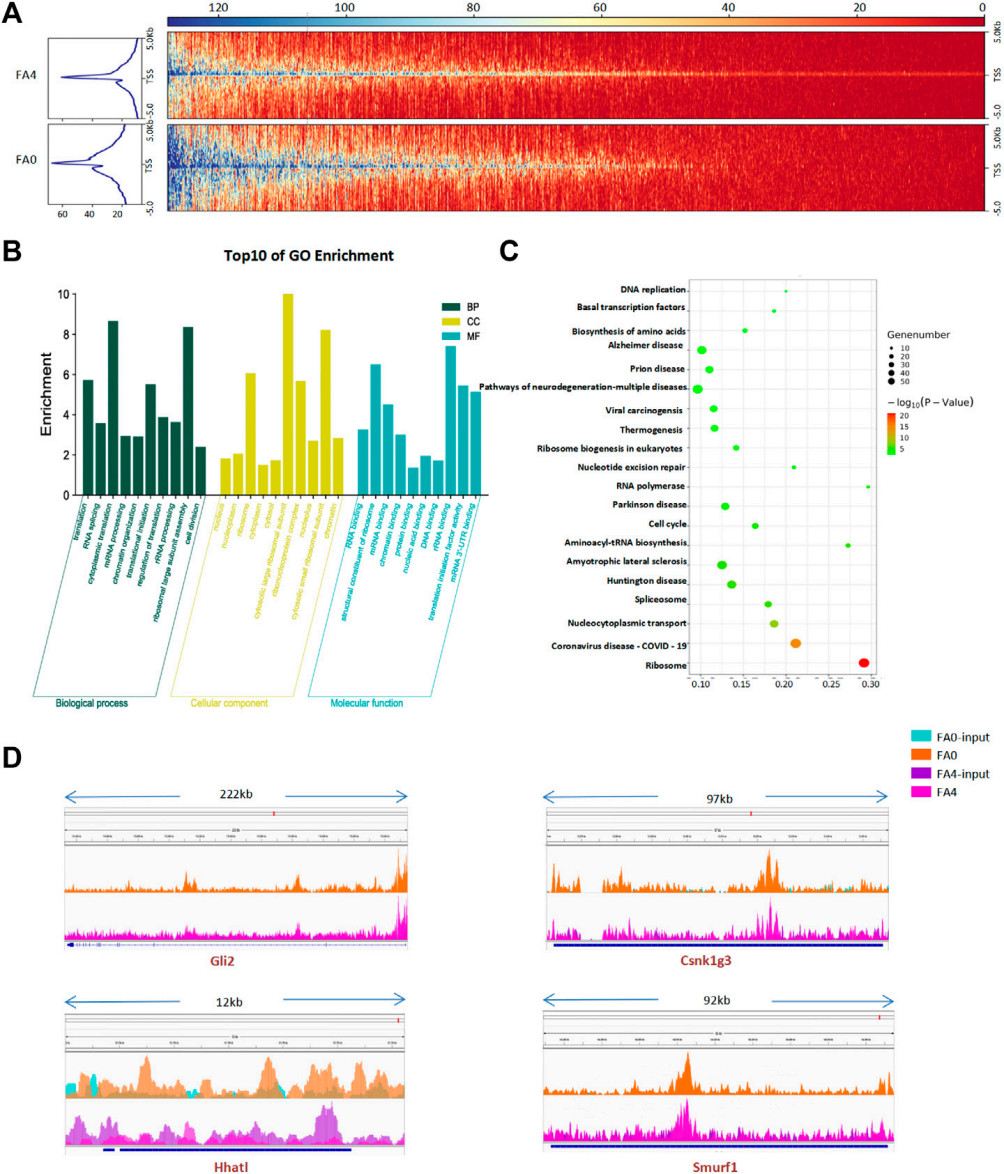
**(A)** Comparison of average ChIP-Seq reads densities and enrichment profiles for H3K27ac in FA (0 mg/L) vs. FA (4 mg/L) mESC. **(B)**. Functional network of enriched genes with H3K27ac enrichment genes in FA (0 mg/L) vs. FA (4 mg/L) mESC. DAVID method was used to do functional annotation clustering for biological process annotations. **(C)** KEGG enrichment analysis of H3K27ac enrichment genes in FA (0 mg/L) vs. FA (4 mg/L) mESC. (Fisher’s exact test; *p* < 0.05). The most popular paths are shown in each category. **(D)** ChIP-Seq density profiles for H3K27ac enrichment on Shh-related to genes (Gli2, Hhatl, Smurf1, Csnk1g3) in FA (0 mg/L) vs. FA (4 mg/L) mESC by IGV.

Epigenetics involves dynamic regulation of DNA methylation and histone modifications to affect gene expression. To further investigate the role of histone H3K27ac in nervous system development, ChIP-seq was performed using folate-deficient mESCs. Analysis of an equal numbers of reads from H3K27ac scanned in the entire mouse genome detected 73,769 peaks across 12,026 genes using anti-H3K27ac antibodies (Supplementary Table S26 and Supplementary Table S27). H3K27ac target genes in ChIP-seq revealed slightly increased levels of H3K27ac accumulation compared with controls in mESCs with folate deficiency. ChIP-seq analysis identified a remarkable overall change in H3K27ac near TSSs in folate-deficient cells (Figure 7A, Supplementary Table S28). H3K27ac enrichment peaks in chromosomes were shown in Supplementary Figure S3A. The Further Gene Ontology (GO) analysis indicated that these differentially expressed genes were enriched in GO terms of multiple biological processes, molecular functions, and cell component (Figure 7B, Supplementary Table S29–S31). In a ChIP-KEGG assay, the top 10 groups with enriched peaks among genes targeted by H3K27ac were related to neurodegeneration pathways (Figure 7C, Supplementary Tables S32). Genes with the highest peaks were associated with nervous system development, followed by neurons and neurogenesis. We calculated the total number of peaks of Shh genes that overlapped between modifications. ChIP-seq analysis showed that H3K27ac enrichment peaks levels of Shh-related genes between control mESC and folate-deficient (Figure 7D). H3K27ac enrichment peaks of some other core genes (*Boc*, *Gas1*, *Cdon*, *Smo*, *Shh*, *Gli1*, *Gli3*) in Shh pathway were shown in Supplementary Figure S3B.

### MeDIP-Seq, hMEDIP-seq and H3K27ac ChIP analysis identifies Shh target genes under folate deficiency in mESC

**FIGURE 8:**
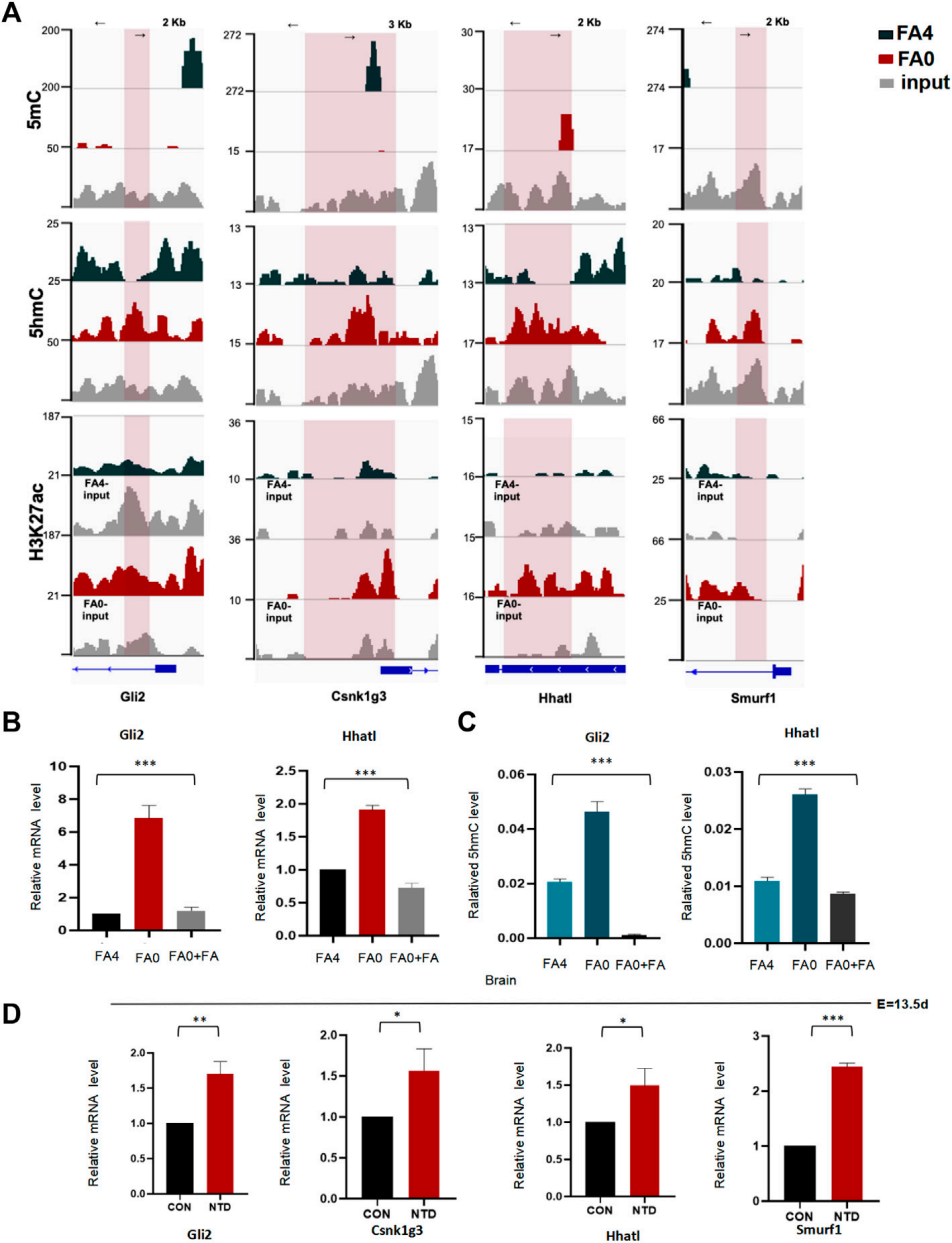
**(A)** Snapshots of the promoter regions of 5hmC, 5mC and H3K27ac profiles of Shh-related to genes (*Gli2*, *Hhatl*, *Smurf1*, *Csnk1g3*). in FA (0 mg/L) vs. FA (4 mg/L) mESC Tracks were shown in IGV. **(B)** Expression levels of Shh-related to genes (*Gli2*, *Hhatl)* in mESCs cultured in in FA (0 mg/L) vs. FA (4 mg/L) and folic acid supplementation mESC determined by RT-qPCR. Data are shown as mean (*n* = 3). *p < 0.05, **p < 0.01, by Student’s t test. **(C)** The 5hmC level of the Shh-related to genes (*Gli2*, *Hhatl*) expressed significant differences in FA (0 mg/L) vs. FA (4 mg/L) and folic acid supplementation mESCs. **(D)** Shh-related to genes (*Gli2*, *Hhatl*, *Smurf1*, *Csnk1g3*) extracted mRNA in cranial neural tissue of MTX-induced mouse NTDs was measured by RT-qPCR. Data are mean ± S. D (*n* = 3). *p < 0.05, **p < 0.01, ***p < 0.001, by Student’s t test.

Several studies have assessed the regulatory relationship between active DNA demethylation and histone modifications such as H3K4me3 and H3K27ac. To correlate DNA demethylation and histone modifications, we compared 5hmC, 5mC, and H3K27ac distributions promoter under folate deficiency. Global assessment of the total binding events between normal folate and folate deficiency demonstrated that a proportion of 5hmC binding events was lost. Some 5hmC binding sites overlapped with 5hmC- and H3K27ac-modified regions, suggesting that TET binds to the gene and catalyzes the hydroxylation of 5-methylcytosine (5mC) to 5-hydroxylmethylcytosine (5hmC) in regulatory elements upon folate deficiency. 5hmC peaks in Shh genes partly colocalized with H3K27ac modification, a histone marker associated with a transcriptionally active state (Figure 8A). As previously reported, the majority of H3K27ac was near TSSs. Interestingly, H3K27ac preferentially overlapped with 5hmC rather than 5mC. Moreover, 5hmC-binding profiles in representative Shh genes were associated with activation of the histone mark H3K27ac (Figure 8A), indicating a transcriptional activation role of folate deficiency in addition to its DNA methylcytosine dioxygenase activity in regulation of Shh gene transcription. Thus, a minority of these two modifications was overlapped in Shh genes. Collectively, although active DNA demethylation correlated to H3K27ac enhancement, this relationship appeared to be limited to certain regions of Shh gene loci. We also examined the expression of Shh pathway genes under folate deficiency in mESCs. Several Shh-related genes were upregulated in folate-deficient mESCs, including Shh genes *Gli2*, and *Hhatl* (Figure 8B). Interestingly, this effect was reversed by folinic acid supplementation (Figure 8B). hMeDIP-PCR revealed that binding of *Gli2* and *Hhatl* to 5hmC was also reversed by folinic acid supplementation (Figure 8C). We then analyzed Shh-related genes in NTD samples from mouse embryos. RT-qPCR showed that the mRNA levels of Shh genes were obviously increased in NTD embryos compared with controls (Figure 8D; *p* < 0.05). *Gli2* belong to the core genes in NTD development. Taken together, these results suggested that *Tet1* levels were increased concomitantly with a positive correlation to Shh gene expression in folate deficiency-induced mouse NTDs.

## Discussion

Several epigenetic studies have suggested a link between an impaired methylation cycle and human NTDs, such as an association with elevated homocysteine and suboptimal levels of folate in maternal blood (Zhang et al., 2018). Folate is absorbed and converted into tetrahydrofolate, which is the carrier of the one-carbon unit, the biologically active form of folic acid. The one-carbon unit for raw material synthesis of pyrimidines and purines plays a pivotal role in the biosynthesis of pyrimidines, purines, protein, and nucleic acids as well as growth and cell division (Hussain, 2012). SAM as a universal one-carbon donor is required for DNA methylation, which is acquired from methyl groups derived from folate (Irwin et al., 2016). Several studies have demonstrated the role of the folate pathway in DNA methylation, but whether the folate deficiency stress-induced hydroxymethylated DNA response is yet to be elucidated. Our data suggested that the genome-wide 5hmC profile was involved in pathways that are significantly associated with folate deficiency. Genome-wide enrichment of the 5hmC modification was found on active TSS and a decrease of 5mC binding to TSS under folate deficiency was observed in mESCs (Figure 3). In our previous study, we found that low global DNA methylation and low 5mC as a mid-product in fetal brain tissue with NTDs was related to NTD-affected pregnancy (Chen et al., 2010). It is worth mentioning that hydroxymethylated DNA is involved in folate metabolism. However, we found that aberrant folate metabolism potentially affected hydroxymethylated DNA. dTTP caused by a lack of folic acid synthesis, dUMP accumulation, and infiltration of DNA results in a decline in the stability of single and double chain rupture and chromatin DNA, which inhibits nucleic acid synthesis. In such cases, cells do not produce enough DNA for cell division, which seriously influences neural tube closure and fetal growth and development, leading to the occurrence of birth defects. Defects in DNA repair often led to neurodevelopmental and neurodegenerative diseases, indicating the importance of DNA repair in post-mitotic neurons. The cycle of cytosine methylation and demethylation on the enhancer of neurons is a potential source of site-specific DNA single-stranded breaks in neurons (Blount et al., 1997; Cali et al., 2019; Wu et al., 2021). Recent studies have also suggested that demethylation of DNA is indirect with modification of the methyl-cytosine by deamination or oxidation, sometimes referred to as ‘hydroxymethlyation’, followed by DNA repair (Greene et al., 2011). Our previous study demonstrated that epigenetic control can be changed by folate deficiency, giving rise to regulation of rRNA gene transcription that causes DNA DSBs (Xie et al., 2017). Whether 5hmC is involved in low-folate NTD fetuses remains to be investigated, which may contribute to our knowledge of the spatiotemporal complexity of nervous system diseases.

The formation and closure of the neural tube is complex, and is precisely regulated by a plenty of developmental regulators during early embryonic development (Saitsu, 2017; Pei et al., 2019; Yu et al., 2019; Heusinkveld et al., 2021). After implantation, total genome reassignment methylation at early fetal development. In recent years, a series of reports has shown that dynamic changes in DNA methylation and the synergistic effect of transcription factors play an important role in the regulation of gene expression (Lin et al., 2017; Hindorff et al., 2018; Luo et al., 2018). The histone acetyltransferase p300 as a transcriptional coactivator regulates gene transcription though acetylated histones and transcription factors in embryonic development and embryonic stem cell differentiation. A study found that the super enhancers of p300 recruitment, such as H3K27ac and H3 at lysine 4 (H3K4me1), may help to identify and regulate the critical point during cell fate decision (Creyghton et al., 2010; Rada-Iglesias et al., 2011). When DNA methylation levels are reduced, the structure of chromosomes is open, which promotes gene transcription. The distal regulatory regions of genes acquire specific activation-enhancing marks accompanied by the appearance of active chromatin of p300, H3K4me1, and H3K27ac (Xie et al., 2013). To select potential epigenetically altered genes, we focused on the Shh pathway. Our present data demonstrated that folate deficiency promoted 5hmC enrichment enhancer H3K27ac binding to Shh pathway genes *Gli2*, and *Hhatl* in mESCs. We found that folate deficiency induced gain of 5hmC *Gli2*, and Hhatl regions. These folate deficiency-induced 5hmC gains are significantly involved in pathways associated with NTDs, such as the p53 signaling pathway, and influence the expression of genes associated with neurodevelopment. Shh signaling involved in regulation of neural plate bending in the mouse embryo development. The Shh signaling pathway is a hedgehog signaling pathway that participates in patterning the dorsoventral axis of the neural tube (Murdoch and Copp, 2010). Three starting points of neural tube closure are different in the position and morphology of neural induction (Bear et al., 2012). Mouse models show that activation of the Shh pathway is associated with NTDs (Murdoch and Copp, 2010). Heterozygous loss-of-function mutations in the Shh signaling lead to abnormal early brain development, such as negative regulators of Shh signaling *Ptch1*, *Pka,* and *Rab23* lead to increased activity of the Shh pathway, which may be associated with exencephaly (Huang et al., 2002; Wang et al., 2013; Hor et al., 2018). In our study, folate metabolism was specifically activated selected Shh-related genes (*Gli2*, and *Hhatl*) in the low-folate brain of NTD mice (Figure 8A). The upregulation of these Shh-related genes by 5hmC may be a molecular regulatory event involved in NTC. This study suggested that 5hmC activates NTC-related genes in folate-deficient mESCs (Figures 5C,D). Folic acid supplementation may attenuate 5hmC and subsequently decrease 5hmC active marks on Shh-related genes at their promoter regions, resulting in decreased expression of Shh-related genes. Folic acid supplementation may also attenuate 5hmC enrichment on Shh and subsequently decrease 5hmC active marks on Shh-related genes at their promoter regions, resulting in decreased expression of NTC-related genes.

Hence, these marks collaborate to affect transcription of genes that regulate NTD development. Folate is particularly important because altered patterns of DNA methylation regulate gene functions and stability, which is termed “epigenetic” modification (Meethal et al., 2013). DNA methylation leads to an “erase-and-rebuild” strategy on a genome-wide scale, resulting in changes in DNA methylation levels (Guo et al., 2014). In a previous study, the global DNA methylation levels in brain tissue of deficient embryos was much lower than that in control embryos, which increased the risk of NTDs (Chen et al., 2010). TET enzymes are the writers of 5hmC status and play an important role in different biological processes. TET-mediated DNA demethylation and the corresponding dynamic regulation of DNA methylation play an important role in embryonic nervous system development (Santiago et al., 2014). Previous studies show that although *Tet1* and *Tet2* despite some overlaps, play distinct roles in mESC re-programming neurodifferentiation and development. Loss of function Tet1 leads to a reduction of the self-renewal capability in adult neuronal stem cells differentiation (Cheng et al., 2018). Double knockout of *Tet1/2* increases mortality in perinatal mice, which is accompanied by external brain malformation, cerebral hemorrhage, and growth retardation (Dawlaty et al., 2013). Triple deletion of *Tet1/2/3* can lead to differentiation defects of neuroectoderm and ectoderm in mice (Li et al., 2016). This suggests that TET gene expression is associated with neural development. We found the different trend in *Tet1*, *Tet2,* and *Tet3* expression under folate deficiency, indicating that TET enzymes have distinct roles in the regulation of neurogenesis. A limitation of our study was that only mouse NTD was collected and low folate human cases are needed to analyze the correlation of Tet levels with 5hmC affecting NTC-gene expression in human NTDs.

Taken together, our results provide strong evidence that folate deficiency induces aberrant 5hmC modification, which is linked to abnormal expression of Shh-related genes and subsequently NTDs. This study extends our understanding of aberrant epigenetic modification of NTC-related genes in NTDs.

## Materials and methods

### Animals

Six-to 8-week-old female and male C57BL/6 mice were purchased from Beijing Vital River Laboratory Animal Technology. Mice were kept under specific pathogen-free conditions with a standard light–dark cycle. Female mice were fed a low folate diet and male mice were fed a normal diet for more than 4 weeks. Temporary mating was achieved by nighttime mating on embryonic day 0.5 (E0.5) when the vaginal lid was open in the morning. Establishment of the NTD mouse model was performed by intraperitoneal injection of 1.5 mg/kg (body weight) MTX (Sigma) at E7.5. Pregnant mice were sacrificed at 13.5 days after mating and embryos were isolated. The embryos were rinsed with pre-cooled DEPC-treated PBS. All procedures, including animal handling, followed institutional guidelines for the care of laboratory animals.

### Detection of maternal serum FA concentration

Mouse FA ELISA Kit (CUSABIO) was used to detect the level of FA in maternal serum. The maternal serum had been obtain by 3000rpm, 15min, 4°C in advance. According to the manufacturer′s protocol, the levels of FA in control maternal serum were compared with those serum which from pregnant mice with NTDs.

### Immunohistochemistry

Fetal mouse brain tissues were fully fixed with 4% paraformaldehyde. After 48 h, these tissues were washed by PBS, dehydrated by ethanol, paraffin-embedded, sliced and washing again. Subsequently, these tissues were dissolved in ethanol and washing. The tissue antigen was repaired. Blocking was using 5% goat serum. TET1 (dilution 1:100; Genetex), TET2 (dilution 1:100; Genetex), TET3 (dilution 1:100; Genetex), DNMT1(dilution 1:100; Genetex), DNMT3b (dilution 1:100; Genetex) as the primary antibodies was performed at 4 °C overnight. Quality was assessed on each batch of slides including a negative control. And the primary antibody was substituted by 10% normal goat serum in order to rule out non-specific signals.

### Cell culture and folic acid treatment

Mouse embryonic stem cell (mESC) line Sv129 was obtained from the Capital Institute of Pediatrics Cell Bank. The cell line of folic acid deficiency group was cultured in Dulbecco’s modified Eagle’s medium (Gibco) supplemented with 15% fetal bovine serum (Gibco), 0.1 mM non-essential amino acids (Gibco), 0.1 mM glutamate (Gibco), 0.07 mM glucose, penicillin (Gibco), 0.1 mM β-mercaptoethanol (Invitrogen), and mouse leukemia inhibitory factor (Millipore). Folate (Sigma) was added to the medium of FA0 as medium of the normal group (final concentration: 4 mg/L). After resuscitating normal mESCs, Sv129 cells were randomly split into FA4 and FA0. Next, two groups were added correspond medium and were cultured at 37°C with 5% CO2 in a humidified incubator and passaged every 2–3 days. The culture medium was changed daily. Folic acid supplementation mESCs should be add FA (50 μM) into FA0 for 24 h.

### RNA-seq and data analysis

Sv/129 cells was used to manufactured as total RNA which selected TRIzol reagent (Invitrogen). Total RNA quality was verified using the Agilent 2100 BioAnalyzer. Library of RNA-sequencing was created using certified total RNA. The NEBNext Ultra RNA Library Prep Kit for llumina was used to build the RNA sequencing library according to manufacturer’s protocol. PCR enrichment and purification of PCR contributed to eatablish final library, then assessed by Agilent 2100 BioAnalyzer. Next, raw data was created using bcl2fastq software from which the original image files of sequencing were identified and were transformed to sequenced reads. Clean data was filtered from raw data. It was aligned to the reference sequence using Hisat2/Tophat. In this step, it was based on all subsequent analysis. Transcript assembly was performed using Stringtie software. The expression level from readcount and the FPKMs of geneswas were then calculated. Differentially expressed gene (DEG) analysis and other bioinformatics analysis were counted using limma/DESeq software. Genes with |Log2 fold change (Log2FC)| ≥1 and *p*-Value ≤ 0.05 were considered as DEGs in comparative analysis. Genes that did not reach these threshold parameters were not considered in further analysis.

### Chromatin immunoprecipitation-seq

Sv129 cells were collected and prepared as ChIP samples using a Simple ChIP Enzymatic chromatin IP kit (9005, CST) in accordance with the manufacturer’s protocol. The DNA concentration in samples was appropriate between 50 and 200 μg/ml. DNA was digested to lengths of 200–500 bp. Anti-H3K27ac (CST) was added to the chromatin preparation to immunoprecipitate chromatin complexes. Quality control of IP samples conducted by an Agilent 2200 before ChIP-sequencing.

### hMeDIP-seq and MeDIP-seq

Before hMeDIP-seq and MeDIP-seq, DNA was extracted from Sv129 cells using a TIANamp Genomic DNA Kit (TIANGEN BIOTECH, China) and fragmented by sonication to yield fragments ranging from 200 to 600 bp. The DNA fragments were captured using a hMeDIP Kit (Active Motif, United States of America) and MeDIP Kit (Active Motif). Extracted DNA was used to build hMeDIP and MeDIP sequencing libraries using a NEBNext Ultra DNA Library Prep Kit for Illumina. Adaptor-ligated DNA was selected by size and enriched by PCR following the manufacturer’s protocol. hMeDIP DNA libraries were quantified using a Quant-iT PicoGreen dsDNA Kit (Life Technologies) and subjected to high-throughput 150 base paired-end sequencing on an Illumina HiSeq sequencer in accordance with the manufacturer’s protocol. Total peaks for genes were detected across the mouse genome by analyzing equal reads at 5mC. By scanning the entire mouse genome, the number of reads and peak splits of genes in 5hmC were detected (fold change ≥1.5, *p* ≤ 0.05).

### hMeDIP-qPCR

DNA samples were fragmented to 200–600 bp. Each sample was divided into two portions: one was enriched as an IP sample by immunoprecipitation with specific antibodies and the other was used as input. DNA samples were purified and subjected to hMeDIP-qPCR verification of target genes. Target gene location information is available in hMeDIP-seq and appropriate primer sequences are available on the UCSC genome browser website (http://genome.ucsc.edu/cgi-bin/hgGateway). Primer sequences are shown in Supplementary Table S33.

### RT-qPCR

Trizol reagent (Invitrogen, United States of America) was used to extract total RNA. Reverse transcription was carried out using BlasTaq 2X Qpcr MasterMix (ABM, Canada). qPCR was performed using Maxima SYBR Green/ROX qPCR Master Mix (ABM). Add one cDNA template to each reaction. Primer sequences are shown in Supplementary Table S33.

### Statistical analysis

All experimental results were presented as means ± SD, which were repeated at least 3 times. The software of GraphPad Prism 8 version was used to analyse all data. A *p*-value of <0.05 was programmed to prompt that data have statistically significant.

## Data Availability

The original contributions presented in the study are publicly available. This data can be found here: NCBI, under the following Accession Numbers: GSE204874, GSE204869, GSE204870, GSE204871 and GSE204872.
