# Supplementary figures and images for: Abnormal transcriptome-wide DNA demethylation induced by folate deficiency causes neural tube defects
Source: Front Genet. 2022 Sep 19;13:987210. doi: 10.3389/fgene.2022.987210 (PMC9529027; doi:10.3389/fgene.2022.987210)

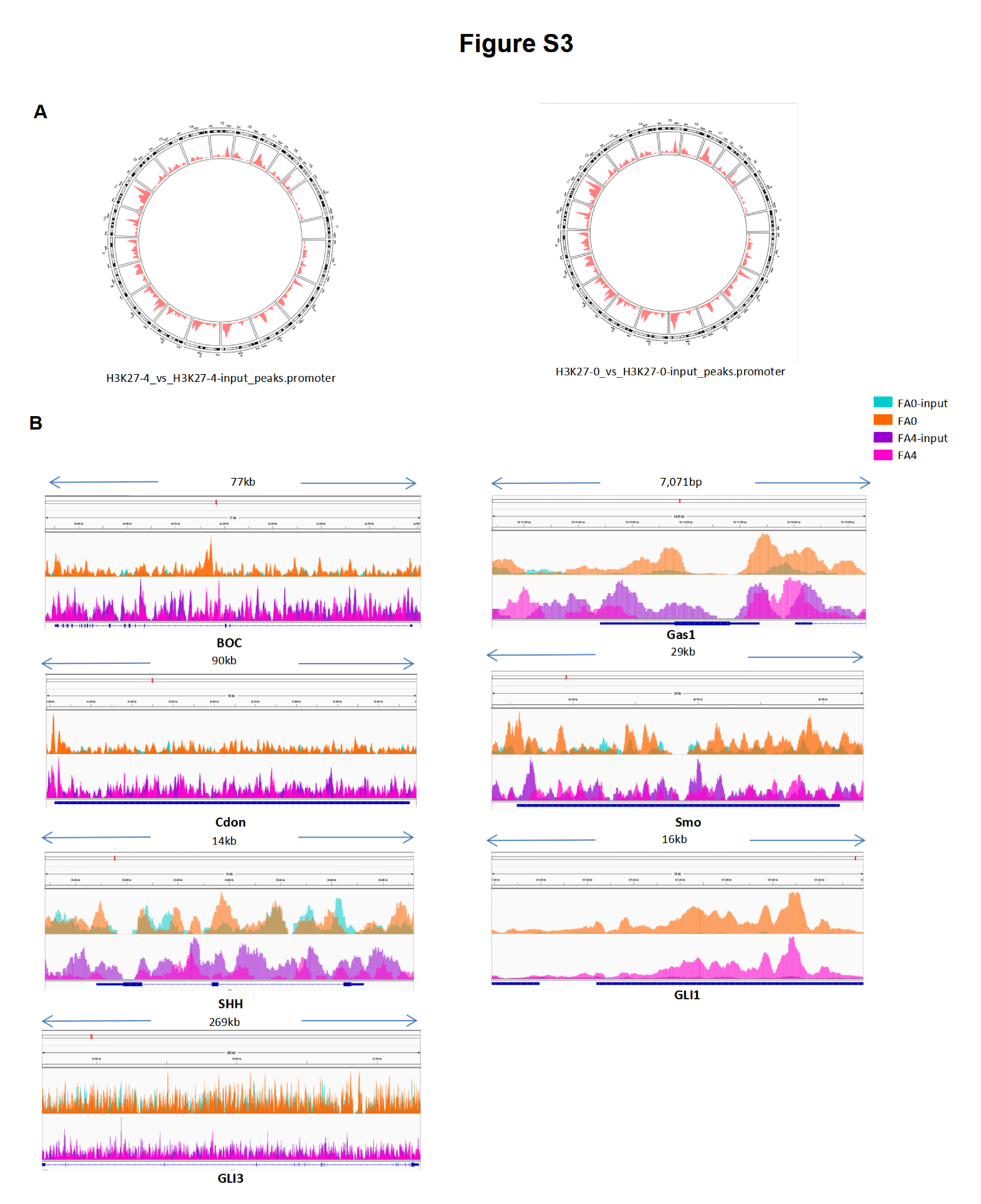

Supplement: Supplementary file 4 [file Image3.jpg]

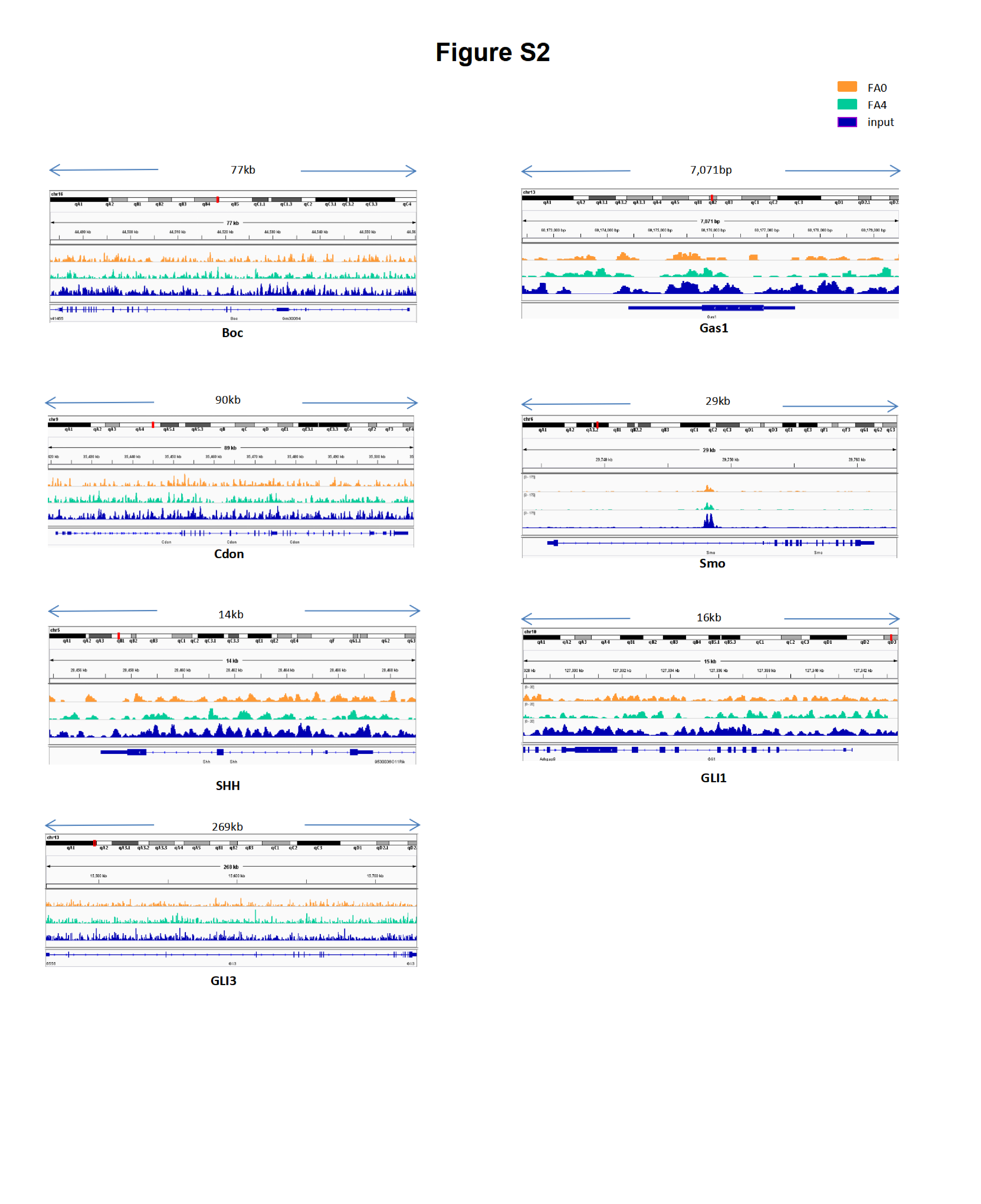

Supplement: Supplementary file 5 [file Image2.jpg]

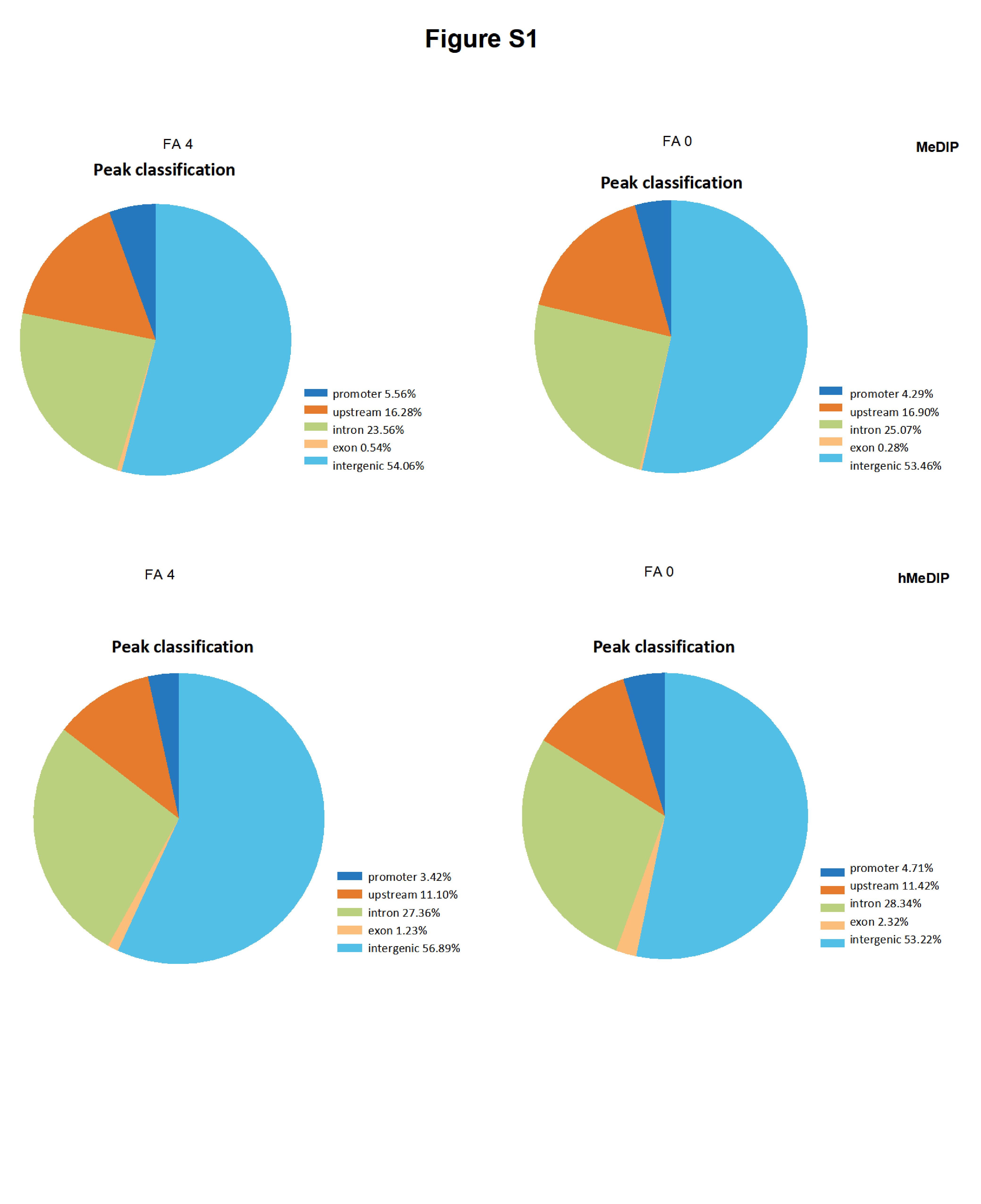

Supplement: Supplementary file 17 [file Image1.jpg]
